# Characteristics of the Chemical Components of PM_2.5_ in the Dangjin Region, South Korea, and Evaluation of Emission Source Contributions During High-Concentration Events

**DOI:** 10.3390/toxics13100869

**Published:** 2025-10-13

**Authors:** Young-hyun Kim, Shin-Young Park, Hyeok Jang, Ji-Eun Moon, Cheol-Min Lee

**Affiliations:** 1 Department of Chemical and Environmental Engineering, Seokyeong University, Seoul 02713, Republic of Korea; rladudgus128@skuniv.ac.kr (Y.-h.K.); tlsdud060900@skuniv.ac.kr (S.-Y.P.); amer1can@skuniv.ac.kr (H.J.); mje0313@skuniv.ac.kr (J.-E.M.); 2 Department of Nano, Chemical and Biological Engineering, Seokyeong University, Seoul 02713, Republic of Korea

**Keywords:** PM_2.5_ concentration, heavy metals, carbonaceous species, water-soluble ions

## Abstract

Fine particulate matter (PM_2.5_; aerodynamic diameter ≤ 2.5 µm) remains a challenging policy for industrialized coastal regions throughout East Asia. In this study, we present a multi-year chemical characterization of PM_2.5_ and identify key factors contributing to extreme pollution events in Dangjin, a heavy-industry hub on Korea’s west coast. Between August 2020 and March 2024, 24-h gravimetric filters (up to *n* = 245; 127–280 valid analyses depending on constituent) were collected twice weekly in winter–spring and weekly in summer–autumn. Meteorological data and 48-h backward HYSPLIT trajectories guided source interpretation. The mean PM_2.5_ concentration was 26.22 ± 15.29 µg/m^3^ (4.74–95.31 µg/m^3^). The mass was highest in winter (30.83 µg/m^3^). Secondary inorganic ions constituted 60.3% of the aerosol, with nitrate comprising 29.7%. A nitrate-to-sulfate ratio of 1.94 indicated a stronger influence from mobile NO_x_ emissions compared to that from coal combustion. The trajectory analysis showed north-easterly transport from Eastern China, followed by local stagnation, which promoted rapid ammonium-nitrate formation. Regional transport contributes to severe PM_2.5_ episodes, with their magnitude increased by local NO_x_ and NH_3_ emissions. Our findings suggest that effective mitigation strategies in coastal industrial corridors require coordinated control of long-range transport and domestic measures focused on vehicles and ammonia-rich industries.

## 1. Introduction

PM_2.5_ has been regulated in Korea under national air quality standards since 2015. PM_2.5_ with an aerodynamic diameter of <2.5 μm, which is smaller than that of PM_10_, can penetrate deep into the lungs and exacerbate respiratory diseases such as asthma, as well as increase the risk of premature death owing to cardiovascular complications [1,2]. The World Health Organization has categorized PM_2.5_ as a Group 1 carcinogen, posing a serious threat to public health [3]. Additionally, PM_2.5_ has a relatively high number of particles per unit volume and a large surface area, facilitating the adsorption of various chemical substances, such as heavy metals, ionic species, and carbonaceous components. Toxic elements such as Cd and As, which have adverse health effects, can be introduced into the human body via PM_2.5_ and may result in various negative health outcomes [4].

The primary chemical constituents of PM_2.5_ comprise water-soluble ions (SO_4_^2−^, NO_3_^−^, and NH_4_^+^), carbonaceous species (organic and elemental carbon), and metals (Fe, Pb, and Cr) [5]. The composition and concentration of these constituents vary based on the characteristics of proximal emission sources [6].

In South Korea, high PM_2.5_ concentrations frequently result from domestic sources and long-range transported aerosols outside the country [7,8]. Typically, foreign contributions are estimated to account for 30–50% of the annual average concentration and up to 60–80% during high-concentration episodes. A joint study by the National Institute of Environmental Research of Korea, China, and Japan estimated that domestic sources contribute approximately 51.2% to annual PM_2.5_ levels in Korea. In contrast, China and Japan contribute 32.1% and 1.5%, respectively [9].

Dangjin, a heavy industry hub on Korea’s west coast, has several primary sources, such as coal-fired power plants, industrial complexes, and steel facilities near residential and commercial areas. Consequently, local emissions considerably influence particulate matter levels, and high-concentration events have also been observed owing to long-range transport from China. Additionally, according to the 2022 Air Quality Annual Report published by the National Institute of Environmental Research, Dangjin, which is situated in the Chungnam region, exhibits the highest average PM_10_ and PM_2.5_ concentrations in the province. Consequently, numerous studies have been conducted on particulate matter’s spatial and temporal distributions and source identification to develop effective control strategies [10,11,12].

We investigated the PM_2.5_ concentrations and their chemical components in Dangjin over 3 years and 7 months, from August 2020 to March 2024. We characterized seasonal variations and compared our findings with previous studies to identify the distinctive features of PM_2.5_ concentrations in Dangjin. Additionally, through multifaceted analyses during high-concentration events, we explored the influencing factors to obtain foundational data and inform future PM_2.5_ management strategies and policy developments in the region.

## 2. Materials and Methods

### 2.1. Monitoring Site

Dangjin is an industrial region with a national industrial complex and several general industrial complexes, 13 comprising the Seongmun National Industrial Complex, which covers 12.0 km^2^. However, part of the Asan National Industrial Complex is officially located in Asan, which borders Dangjin. In particular, the general industrial complexes are also extensive; however, the national industrial complexes alone cover approximately 16,877 m^2^ [13]. To investigate the distribution characteristics of PM_2.5_ and its chemical components in Dangjin’s ambient air, we selected a monitoring site in a residential area close to major industrial facilities.

**Figure 1:**
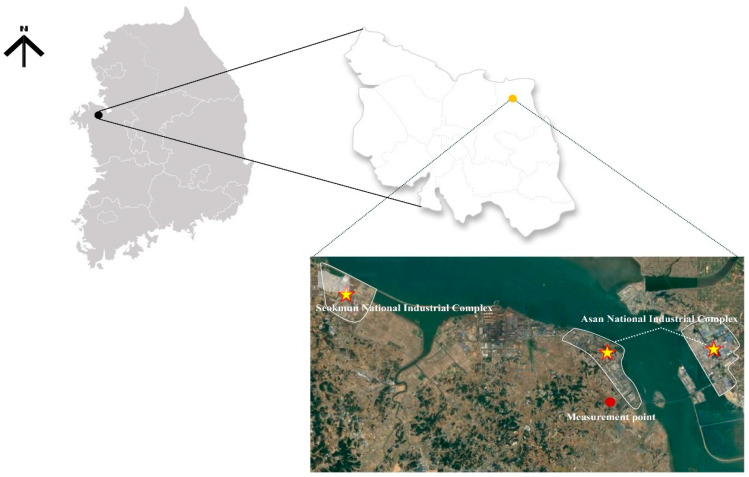
Air monitoring stations within the Dangjin industrial complex. Background map source: Google Maps https://www.google.com/maps (accessed on 10 April 2025).

The air monitoring site was set at coordinates (36°56′30.4″ N, 126°47′04.1″ E) to represent the air quality of both industrial and residential areas in central Dangjin (Figure 1). To ensure an accurate representation of the local air quality without notable nearby interference, samples were collected from a building rooftop presumed to be minimally affected by immediate surrounding sources.

As of July 2024, 496 companies were operating within Dangjin’s industrial complexes. Of these, 200 companies were in the Seongmun Industrial Complex (one of the national industrial complexes), and 142 companies were in the Godae-Bugok District of the Asan National Industrial Complex, which partially extends into Dangjin. The operational rates for these complexes were 46% and 95.7%, respectively. Machinery, petrochemicals, and steel accounted for approximately 76% of the industries within the Seongmun Industrial Complex; other operations included valve manufacturing, metal storage, chemical dye production, and surface treatment. In the Asan Industrial Complex, steel, semiconductors, power generation, and automobile parts manufacturing accounted for approximately 50% of all industries [14].

### 2.2. Sample Collection

This study was conducted over 3 years and 7 months, from August 2020 to March 2024, by selecting measurement points and collecting samples in a national industrial park in Dangjin. Measurements were conducted twice a week (Monday and Wednesday) during winter, which typically has high concentrations of fine particulate matter [15], and during spring, which is frequently affected by yellow dust [16]. During summer and autumn, measurements were conducted once a week (Monday). The dataset and subsequent analyses excluded samples affected by extreme weather events such as typhoons, heavy rainfall, or instrument failure, and the overall sampling strategy followed the protocol described in our previous study [17]. PM_2.5_ was collected using a gravimetric method with a low-volume air sampler (PMS-204, APM Co., Bucheon, Republic of Korea), which conforms to the U.S. EPA Federal Reference Method for PM_2.5_ measurement (Online Resource 1).

Sampling ran continuously for 24 h from 09:00 at a constant flow of 16.7 L/min on each measurement day. All equipment setup, sample acquisition, and transport activities were implemented strictly with the “Guidelines for Installation and Operation of Air Pollution Measurement Networks” provided by the Ministry of Environment and the National Institute of Environmental Research [18]. Two independent sampling devices were employed to facilitate analysis of ionic constituents, trace metals, and carbon fractions. Each device was outfitted with Teflon filters (PTFE, 2.0 μm pore size, Ø 27 mm) and quartz fiber filters (Pure Quartz fiber, Ø 47 mm), following NIER specifications.

The Teflon filters were placed in sterile Petri dishes, sealed with paraffin film, and refrigerated until analysis, and the quartz filters were used after the heating treatment. To prevent the loss of PM_2.5_ components through volatilization and reaction, the collected dust was transported in an ice box filled with refrigerant to maintain a low temperature, and a buffered transport device was used to prevent loss of PM_2.5_ particles owing to shaking or impact during transport, and to prevent static electricity.

### 2.3. Gravimetric Analysis

Each filter was conditioned and weighed for 24 h under controlled temperature and humidity before and after sample collection. Laboratory blanks (LAB) and field blanks (FB) were used to correct the measured PM_2.5_ mass concentrations. For each batch of filters, a new LAB was kept in the laboratory during the sampling period and reweighed post-collection to verify measurement accuracy. Similarly, a fresh FB was transported to the field site without exposure to airflow, then returned and reweighed to assess handling stability. The PM_2.5_ mass concentration was subsequently calculated using the following equation:

(1) $${PM}_{2.5}(µg/m^{3})=\frac{\left(W_{f}-W_{i})-(W_{FBf}-W_{FBi}\right)}{Va}$$

where W_i_ and W_f_ denote the initial and final weights (µg) of the LAB filters; W_FBi_ and W_FBf_ indicate the weights (µg) of the FB filters measured before and after PM_2.5_ sampling; and Va represents the total air volume (m^3^) drawn through the sampler.

### 2.4. Ionic Composition Analysis

The ionic constituents of PM_2.5_ were determined, targeting five cationic (Na^+^, NH_4_^+^, K^+^, Mg^2+^, and Ca^2+^) and three anionic species (SO_4_^2−^, NO_3_^−^, and Cl^−^). To enhance analyte extraction efficiency, the filter was positioned such that its collection surface faced the bottom of a beaker, and 200 μL of ethanol (reagent grade or higher) was added to promote precipitation. Subsequently, 20 mL of ultrapure water (resistivity ≥ 18 MΩ·cm) was introduced, and the solution was stirred at 120 rpm for 120 min using a magnetic stirrer.

Following the extraction, the liquid was passed through a 110 mm diameter membrane or a syringe filter with a 0.1-μm pore size. Afterward, ionic species were quantified using ion chromatography (IC) under conditions presented in Online Resource 2.

Calibration standards were prepared for each target ion, and calibration curves were constructed accordingly. The coefficient of determination (R^2^) for all analytes was >0.999, indicating high analytical linearity. The atmospheric concentrations of ionic species in PM_2.5_ were then computed using the following equation:

(2) $$C=((X_{1}-X_{2})\times S)÷F$$

where C denotes the atmospheric concentration of ionic species (µg/m^3^); X_1_ and X_2_ are the concentrations (µg/mL) in the sample and blank filters, respectively; S indicates the extracted solution volume (mL); and F refers to the total sampled air volume (m^3^).

### 2.5. Carbon Component Analysis

The flame ionization detector was used to analyze the carbon component of PM_2.5_ (Online Resource 3). The collected filters were baked at 550 °C for >4 h to remove impurities such as organic components and cut into 1.5 cm^2^ sample pieces for analysis. To prevent sample contamination owing to the use of sample autoinjectors, a maximum of 10 samples were analyzed consecutively. Additionally, the temperature was maintained at a low level, and the analysis was conducted on days with high humidity. The concentration of carbon in PM_2.5_ was calculated using the following equation:

(3) $$C=((X_{1}-X_{2})\times S)÷F$$

where C denotes the atmospheric concentration of carbon species (µg/m^3^); X_1_ and X_2_ are the surface concentrations (µg/cm^2^) from the sampled and blank filters, respectively; S indicates the analyzed filter area (cm^2^); and F refers to the total volume of air sampled (m^3^).

### 2.6. Trace Elemental Composition Analysis

The trace elemental composition of PM_2.5_ was analyzed using energy-dispersive X-ray fluorescence (ED-XRF). A total of nineteen elements were quantified, including Al, Ti, V, Mn, Fe, Ni, Co, Zn, As, Sr, Mo, Cd, Ba, Pb, P, S, Cr, Si, and Cu. The procedures were carried out in accordance with the national guidelines for air pollution monitoring networks established by the Ministry of Environment and the National Institute of Environmental Research. Technical details of the analytical equipment are provided in Online Resource 4.

ED-XRF, which has a three-dimensional optical system structure, is a quantitative and qualitative analysis instrument that measures the wavelength and intensity of unique fluorescent X-rays emitted by each element and can measure harmful substances in the atmosphere in extremely small amounts. Standard solutions were prepared for the analysis of trace elemental composition. Additionally, calibration curves were generated for each standard substance, and the coefficient of determination (R^2^) was >0.999. The concentration of trace elemental components in PM_2.5_ was calculated using the following equation: the mass of components in 1 m^3^ of air at 0 °C and 760 mmHg.

(4) $$C=\frac{(C_{s}-C_{bk})\times A_{u)}}{V_{s}}$$

where C represents the concentrations (µg/m^3^) of the trace elements in the atmosphere, C_S_ is the mass (ng/cm^2^) of heavy metals collected on the filter, C_bk_ is the mass (ng/cm^2^) of heavy metals on the blank filter, A_u_ is the total area (cm^2^) of the filter used for sampling, and vs. is the total volume (m^3^) of the air sampled.

### 2.7. Quality Control (QA/QC)

To calculate the accurate value of the measured item, precision control was performed once yearly, and the method detection limit (MDL), relative standard deviation, and the coefficient of determination of the black curve (R^2^)’s straightness were calculated for precision control. The MDL is the standard deviation of the measurement multiplied by 3.14, and the relative standard deviation (unit: %) is the standard deviation divided by the mean value of n consecutive measurements and multiplied by 100. Finally, a calibration curve was generated using two or more standards with concentrations within the quantification range, and the coefficient of determination of the straightness of the curve was calculated. If the R^2^ is <0.99, the curve is rewritten. The MDL values ranged from 0.001 (Ag) to 1.547 µg/m^3^ (Sb), and the R^2^ of the calibration curves for each component was >0.99.

### 2.8. Statistical Analysis

This study calculated descriptive statistics such as mean, standard deviation, and minimum and maximum values to determine the spatial and temporal distribution of PM_2.5_ concentrations and chemical constituents in PM_2.5_. Subsequently, Pearson correlation analysis was used to evaluate the correlation between major ionic components (NO_3_^−^, SO_4_^2−^, and NH_4_^+^), carbon components (organic carbon [OC] and elemental carbon [EC]), and trace elements, and one-way analysis of variance was used to test for seasonal and annual differences in concentrations. The significance level for all statistical analyses was set at 0.05. In addition, we identified the causes of high concentrations by comparing the increase in major elements during high-concentration events (PM_2.5_ > 75 µg/m^3^) to normal times.

### 2.9. Weather Information Processing and the Hybrid Single-Particle Lagrangian Integrated Trajectory (HYSPLIT) Model

Meteorological data from Dangjin City were used to conduct atmospheric analysis, based on records obtained from the Automatic Weather System operated by the Korea Meteorological Administration. To determine the origin of increased PM_2.5_ concentrations, backward trajectory simulations were performed using the HYSPLIT model. Developed as part of the Real-time Environmental Applications and Display System by the National Oceanic and Atmospheric Administration, HYSPLIT estimates atmospheric transport pathways based on outputs from numerical weather prediction models.

In this study, meteorological input fields were obtained from the Global Data Assimilation System, operated by the National Centers for Environmental Prediction, with a temporal resolution of 1 h and a spatial resolution of 1°. Backward trajectory simulations were conducted using the HYSPLIT model with meteorological input from the Global Data Assimilation System (GDAS1, 1° × 1°, 1 h resolution). Trajectories were computed for a 72 h backward period, starting at arrival heights of 500, 1000, 2000, and 3000 m above ground level, with additional cases at 5000 m to capture higher-altitude transport. The arrival point was the Dangjin monitoring site (36.99° N, 126.62° E). The model top was set at 10,000 m, with vertical motion fields obtained from the meteorological input data. Trajectory outputs were recorded at 1 h intervals.

High PM_2.5_ episodes were identified based on daily mean concentrations, with exceedances defined as days when concentrations exceeded 75 µg/m^3^, the threshold for issuing PM_2.5_ advisories in Korea. These selected episodes were further assessed using wind rose plots and HYSPLIT simulations to differentiate between transboundary inflow and local emissions, in conjunction with evaluating changes in chemical composition and particle size distribution.

## 3. Results

### 3.1. Meteorological Data

Based on data from the Dangjin Automatic Weather System between August 2020 and March 2024, the average temperature was 12.9 °C, with an average high temperature of 35.1 °C and an average low temperature of −16.2 °C. Cumulative precipitation totaled 1364 mm over the study period. The prevailing wind directions were southerly (39.76%) and northerly (28.65%), indicating that winds predominantly blew from the south or north.

Among the meteorological conditions facilitating air pollution episodes, calm conditions, which are defined as wind speeds of ≤0.5 m/s, accounted for 18.34% of the study period. However, this percentage indicates that wind-free periods were relatively limited, and stagnant air masses associated with high PM_2.5_ days can prolong the residence time of transported particulates [19]. Further, calm conditions may facilitate increases in locally formed secondary particles, potentially increasing PM_2.5_ particle concentrations [20].

### 3.2. PM_2.5_ Seasonal Distribution and Chemical Components

**Figure 2:**
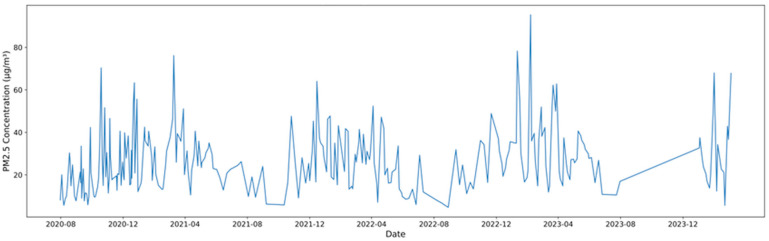
Daily variation of PM_2.5_ mass concentration.

Throughout the study, PM_2.5_ levels exhibited significant variation, ranging from 4.74 to 95.31 μg/m^3^, with a mean value of 26.22 ± 15.29 μg/m^3^. The daily variation of PM_2.5_ concentrations is illustrated in Figure 2, showing distinct fluctuations corresponding to seasonal and meteorological influences. Seasonally, the average concentration in spring was 30.58 ± 14.10 μg/m^3^, which decreased to 15.68 μg/m^3^ in summer, the lowest among the four seasons, before increasing to 22.11 ± 13.56 μg/m^3^ in the fall. Winter had the highest value at 30.83 ± 16.31 μg/m^3^. These findings are consistent with those of previous studies [15,16], indicating relatively high winter concentrations owing to heating emissions, meteorological factors, and springtime increases, which are influenced by Asian dust events. Therefore, the seasonal PM_2.5_ trends observed in Dangjin are consistent with the typical distribution patterns reported in other regions of Korea.

**Figure 3:**
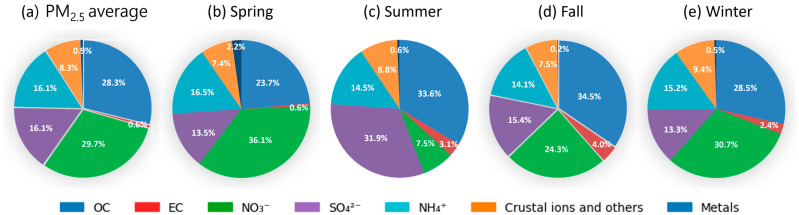
(**a**) Annual and seasonal (**b**–**e**) average chemical compositions of PM_2.5_.

**Figure 4:**
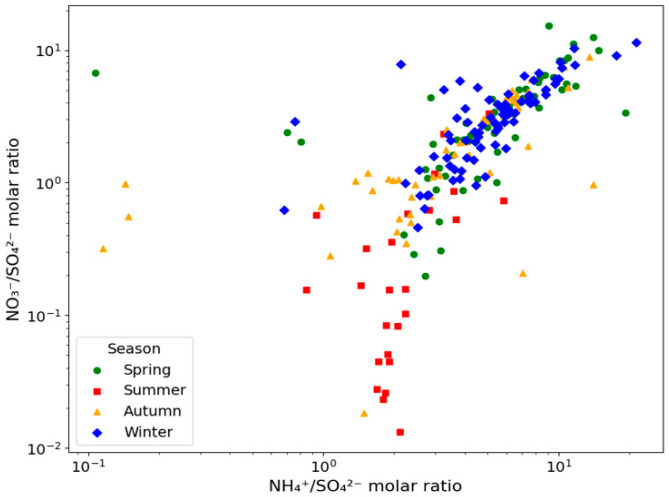
Nitrate-to-sulfate molar ratio as a function of ammonium-to-sulfatemolar ratio in PM_2.5_.

The mean NO_3_^−^ concentration in PM_2.5_ during the monitoring period was 5.69 ± 7.02 μg/m^3^, accounting for the largest proportion at 29.7% of the total PM_2.5_ mass; OC followed this at 28.3% and SO_4_^2−^ and NH_4_^+^ each at 16.1%. The annual and seasonal average chemical compositions of PM_2.5_ are presented in Figure 3. These three secondary inorganic ions collectively comprised more than half (61.9%) of the total PM_2.5_. When the NO_3_^−^/SO_4_^2−^ ratio is >1, mobile emission sources such as vehicular exhaust are more influential, whereas ratios < 1 indicate a considerable effect from stationary sources such as coal combustion [21]. Therefore, with a NO_3_^−^/SO_4_^2−^ ratio of 1.84 in the study area, mobile emissions—particularly those from automobiles—appear to contribute more to PM_2.5_ than from stationary sources. The relationship between the NO_3_^−^/SO_4_^2−^ and NH_4_^+^/SO_4_^2^^−^ ratios is illustrated in Figure 4. Additionally, the NH_4_^+^/SO_4_^2−^ ratio was consistently >1.5 across all seasons, indicating that under ammonia-rich atmospheric conditions in Dangjin, interactions with acidic substances may enhance PM_2.5_ formation [22,23].

Carbonaceous compounds accounted for approximately 28.9% of the total PM_2.5_ mass. This fraction introduces OC into the atmosphere through direct emissions and secondary formation driven by photochemical reactions involving volatile organic compounds (VOCs) [24]. In contrast, EC primarily results from combustion-related processes, including burning fossil fuels and biomass, and is a marker of primary emissions [25]. The interpretation of OC/EC ratios provides insights into emission sources: values < 1 typically indicate coal combustion, whereas ratios > 1 indicate substantial influence from secondary processes, including biomass burning [26,27,28]. An OC/EC ratio ≥ 2.0 is indicative of secondary organic carbon (SOC) formation via atmospheric photochemical transformations [29,30].

This study’s OC/EC ratio was 8.72, indicating that secondary formation was more influential than primary sources; this may be attributed to the local characteristics of the sampling site. The site, which is situated near a major roadway, is considerably affected by vehicular emissions and is surrounded by numerous industrial facilities that emit combustion-related pollutants. Additionally, pollutants generated in adjacent regions can be carried by wind, contributing to increased contamination. These local factors likely promote the accumulation of primary pollutants and facilitate active photochemical reactions, thereby increasing the production of secondary OC.

Although trace elements account for a relatively minor fraction of the total mass, numerous toxic metals within PM_2.5_ can adversely affect human health even at low concentrations [31]. Therefore, continuous monitoring is crucial. This study measured the mass concentrations of 19 trace elements—S, Al, Si, Fe, Zn, Mn, Pb, Ti, Ba, P, As, Cu, Cd, Cr, Mo, Ni, V, Sr, and Co. Their combined contribution was under 10% of the total PM_2.5_, with an annual average concentration of 1.4 μg/m^3^ (Table 1).

The correlation analysis of primary PM_2.5_ components revealed strong inter-component correlations in all seasons except for summer (Table 2). In summer, the correlation coefficient between NO_3_^−^ and SO_4_^2−^ was −0.15, indicating a negative relationship. This finding is consistent with a previous study [32], which revealed that when temperatures exceed 30 °C, atmospheric NO_3_^−^ exists primarily as gaseous HNO_3_. Owing to the increased solar radiation and temperatures in summer, NO_3_^−^ remains in its gaseous form rather than transitioning to the particulate phase; consequently, the resulting inverse correlations among components differ from those in other seasons.

In contrast, in winter, NH_4_^+^ and NO_3_^−^ exhibited the strongest correlation (0.94). As previously mentioned, NO_3_^−^ concentrations were higher in winter than in other seasons, and secondary NO_3_^−^ primarily exists as ammonium nitrate (NH_4_NO_3_).

**Figure 5:**
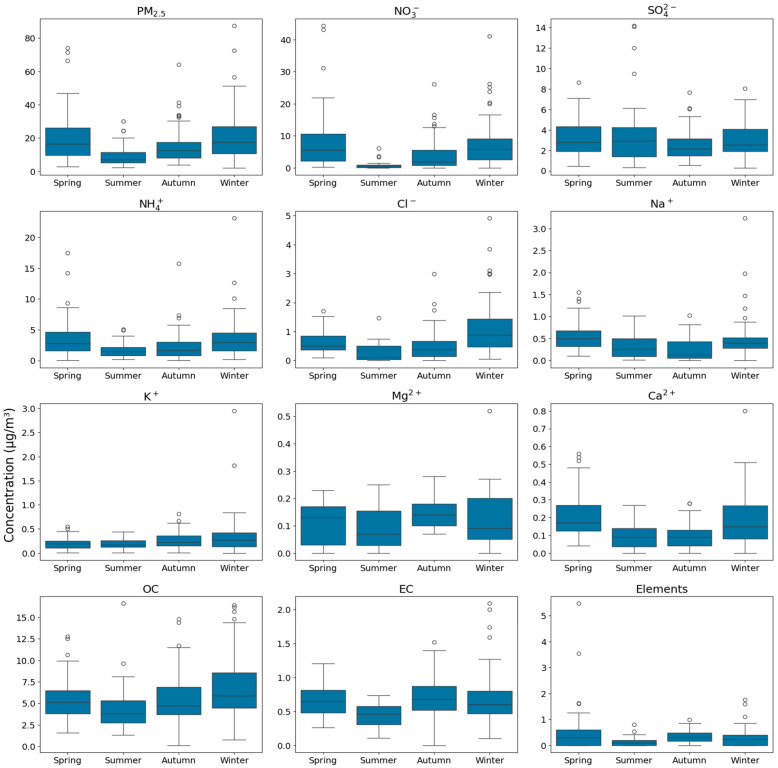
Seasonal variation of PM_2.5_ components.

Figure 5 shows the seasonal concentration distributions of PM_2.5_ and all its chemical constituents. An analysis of each substance by season indicated that PM_2.5_ concentrations were generally the highest in winter and lowest in summer. This outcome can be attributed to coal combustion for heating and stagnant atmospheric conditions in winter and, conversely, to high mixing heights and frequent precipitation events in summer, consistent with previous findings [33]. The seasonal order of PM_2.5_ concentrations was winter > spring > fall > summer, with winter exhibiting marginally higher levels than spring.

Circles represent outliers beyond 1.5 times the interquartile range (IQR). Increased NO_3_^−^ and NH_4_^+^ concentrations were observed in both spring and winter, possibly owing to low-temperature phase changes, long-range transport, and stagnant conditions [34]. In contrast, SO_4_^2−^ was highest during summer, likely influenced by high temperatures, high humidity, intense solar radiation, and a deep mixing layer, all promoting SO_4_^2−^ formation [35].

Cl^−^ averaged 1.07 ± 0.87 μg/m^3^ in winter, exceeding levels in other seasons. Although coarse-mode Cl^−^ is typically associated with marine sources, in fine-mode particles, increased Cl^−^ levels typically result from industrial processes, waste incineration, and coal combustion [36]. Consequently, coal combustion for heating has likely played a notable role in winter. K^+^ and Mg^2+^ concentrations exhibited similar seasonal patterns, remaining relatively low from spring to summer and slightly increasing in the fall. This pattern may be attributed to the lower total PM_2.5_ mass in the fall, which proportionally increases the K^+^ and Mg^2+^ concentrations. These ions commonly originate from marine sources, soil, and biomass burning [37].

Ca^2+^ and Na^+^ concentrations are influenced by soil erosion, resuspension, and marine sources, and are typically found in high quantities in coarse particles [38]. However, Ca^2+^ primarily originates from the soil, whereas Na^+^ predominantly results from marine sources. In spring, strong winds and stagnant air possibly contributed to both ions becoming essential components of PM_2.5_. Spring had the highest proportion of heavy metals, likely due to Asian dust events transporting soil from deserts in China and Mongolia via prevailing westerly winds [39].

### 3.3. Characteristics of High-Concentration PM_2.5_ Components

**Figure 6:**
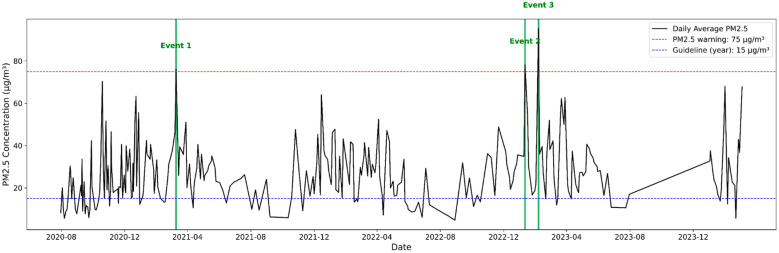
Temporal variations in daily average PM_2.5_.

This study defines high-concentration events as days during which the PM_2.5_ advisory threshold (75 μg/m^3^) is exceeded consecutively for at least 24 h. The temporal variations in daily average PM_2.5_ are shown in Figure 6, highlighting the three identified high-concentration events. Three such high-concentration events were identified and analyzed. To investigate changes in major components during each event, the mean values for the three high-concentration days were compared with those for the seasonal averages, excluding the event days. Among these events, PM_2.5_ concentrations were the highest in Event 3 (95.31 μg/m^3^), followed by Events 2 (78.23 μg/m^3^) and 1 (76.14 μg/m^3^). Notably, concentrations of ammonium (NH_4_^+^), nitrate (NO_3_^−^), sulfate (SO_4_^2−^), and OC significantly increased during these high PM_2.5_ episodes, indicating that these components were key contributors.

**Figure 7:**
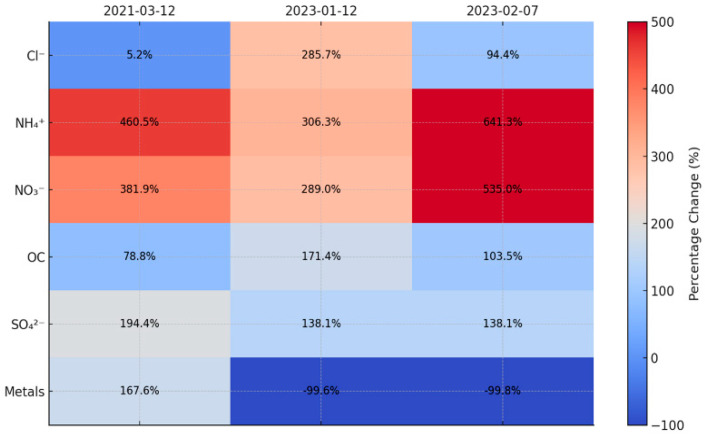
Change Rates of Major Components in High-Concentration PM_2.5_.

The percentage changes of major components during each high-concentration event are shown in Figure 7. Ammonium (NH_4_^+^) exhibited the largest increase relative to its seasonal average during these high-concentration periods: approximately 4.6-, 3.1-, and 6.4-fold increases in Events 1, 2, and 3, respectively. Ammonium typically combines with nitrate (NO_3_^−^) and sulfate (SO_4_^2−^) to form ammonium nitrate (NH_4_NO_3_) and ammonium sulfate ((NH_4_)_2_SO_4_), respectively [40]. Consequently, it plays a major role as a secondary component in high-concentration PM_2.5_. In Event 3, which occurred in winter, the substantially high ammonium concentration further accentuated its linkage to nitrate.

Nitrate (NO_3_^−^) concentrations increased by approximately 3.8-fold in Event 1, 2.9-fold in Event 2, and 5.3-fold in Event 3 relative to seasonal averages. Nitrate is a secondary pollutant formed when nitrogen oxides (NO_x_) undergo oxidation reactions in the atmosphere, originating mainly from mobile sources, particularly vehicle exhaust [41]. The exceptionally high increase during Event 3 possibly indicates a combination of heating (during winter) and vehicular emissions. Moreover, the low mixing-layer height and stagnant conditions in winter likely facilitated nitrate accumulation, thereby amplifying ambient concentrations.

Sulfate (SO_4_^2−^) increased by approximately 2-fold in Event 1 and 1.4-fold in Events 2 and 3 compared to seasonal averages, primarily associated with stationary sources, such as coal combustion [42]. Sulfate is formed secondarily through oxidation reactions of sulfur dioxide (SO_2_) in the atmosphere. Emissions from these stationary sources can increase ambient sulfate concentrations.

OC levels in high-concentration events increased by approximately 0.8-, 1.7-, and 1.0-fold in Events 1, 2, and 3, respectively, compared to seasonal averages. OC is partly formed via the photochemical oxidation of VOCs in the atmosphere and is vital in high PM_2.5_ episodes [43]. This study’s OC/EC ratio was 8.72, indicating that SOC contributes substantially to the total OC, exceeding primary emissions. Low mixing-layer heights and stagnant conditions likely promoted VOC oxidation reactions, further enhancing SOC formation. Additionally, interactions with other components, such as nitrate (NO_3_^−^) and sulfate (SO_4_^2−^), may have contributed to the formation of complex aerosols during these episodes.

## 4. Causes of High-Concentration Occurrences

**Figure 8:**
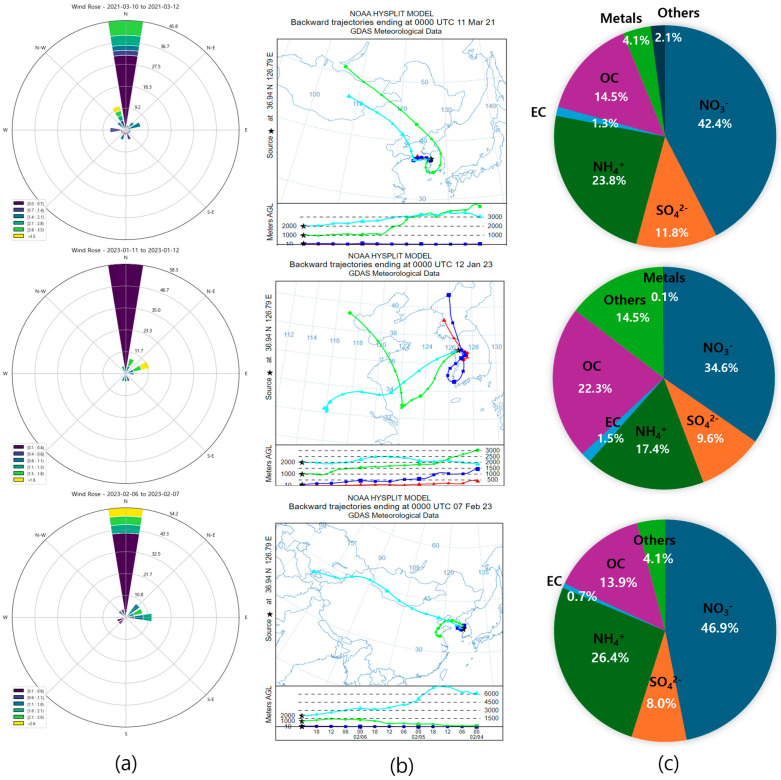
(**a**) Windrose; (**b**) Back-trajectory; (**c**) Chemical composition ratio. In (**b**), colored lines denote air mass trajectories at various arrival heights, and the black star marks the Dangjin monitoring site.

Wind direction and wind speed data are important meteorological factors influencing variations in fine particulate matter concentrations. In particular, the effect on surrounding areas can vary considerably depending on the daily changes in prevailing wind direction [44,45]. This study used wind roses based on wind direction and wind speed data from Dangjin City to examine the influence of surrounding emission sources on fine particle concentrations. Additionally, the prevailing wind direction in each case and the physical and chemical changes of PM_2.5_ were investigated. Figure 8 shows the wind roses, backward trajectory analysis, and physical and chemical changes for three high-conce-tration PM_2.5_ cases measured in Dangjin during the study period.

Case 1 occurred over two consecutive days, from 10 to 12 March 2021, during the spring season. The PM_2.5_ concentration was 76.14 µg/m^3^, with a prevailing northerly wind. The average wind speed was 1.19 m/s, and the calm condition ratio was 35.42%, indicating the highest wind speed and lowest calm condition ratio among the high-concentration cases. The backward trajectory analysis indicated that air parcels likely originated from the northern continental regions of East Asia, gradually ascended to higher altitudes, and subsequently entered Korea. All air masses in the upper and lower layers were tran-ported from northwestern continental areas, contributing to the observed influence. The chemical composition was as follows: NO_3_^−^ (42.4%), NH_4_^+^ (23.8%), OC (14.5%), SO_4_^2−^ (11.8%), metals (4.1%), and EC (1.3%). During this springtime high-concentration case, SO_4_^2−^ was relatively high owing to an increase in SO_2_, a precursor to SO_4_^2−^. The increase in SO_4_^2−^ during spring high-concentration periods may be due to the high levels of SO_2_ (a gaseous precursor) and more active gas-phase reactions under lower relative humidity than those of low-concentration periods in spring. Therefore, this springtime high-concentration event is likely associated with long-range transported air masses from the northern continental regions, as suggested by the backward trajectory analysis.

Case 2 occurred in winter, from 11 to 12 January 2023. The PM_2.5_ concentration was 78.23 µg/m^3^, with a prevailing northerly wind and some observed northwesterly winds. The average wind speed was 0.68 m/s, and the calm condition ratio was 58.8%, the highest among the cases. The significant increase in the concentrations of NO_3_^−^ and NH_4_^+^ in the atmosphere was primarily due to their long residence time, a characteristic pattern particularly associated with winter heating fuel usage. The backward trajectory analysis showed that air masses during this period mainly originated from northern and northeastern continental regions and moved toward Korea, where local stagnation further contributed to the high-concentration event. The chemical composition was as follows: NO_3_^−^ (34.6%), OC (22.3%), NH_4_^+^ (17.4%), SO_4_^2−^ (9.6%), and EC (1.5%). Compared to the normal periods, NO_3_^−^ and NH_4_^+^ concentrations increased by approximately 1.2 and 1.18, respectively, whereas Cl^−^ concentrations increased by a factor of 5.5, which was a notable increase. During this period, heating activities involving coal combustion and chlorides emitted from metal processing and manufacturing processes in the Asan National Industrial Complex were analyzed as the primary causes for increased Cl^−^ levels. Therefore, the combined influence of long-range transported air masses and domestic industrial activities likely contributed to this high-concentration event.

Case 3 also occurred during winter, from 6 to 7 February 2023, with a PM_2.5_ concentration of 95.31 µg/m^3^. The prevailing wind was northerly, and some easterly winds were observed. The average wind speed was 0.92 m/s, and the calm condition ratio was 50.0%, a level that can facilitate local airflow stagnation. The backward trajectory analysis showed that air masses mainly originated from eastern continental regions of East Asia and moved along the west coast before reaching Korea, contributing to the high-concentration event. The chemical composition analysis indicated the following concentrations: NO_3_^−^ (46.9%), NH_4_^+^ (26.4%), OC (13.9%), SO_4_^2−^ (8.0%), and EC (0.8%). The notable increase in NO_3_^−^ can be attributed to low temperatures and high humidity during winter, which promote the partitioning of nitrates into the aerosol phase and enhance heterogeneous reactions under high humidity conditions. The long-range transport of continental air masses and local stagnation were important factors in increasing nitrate levels. These combined factors resulted in the formation of the high-concentration PM_2.5_ event in Case 3.

The backward trajectory analyses in Cases 1–3 were based on the HYSPLIT model, which provides simulated transport pathways. Actual transport may be more complex, and observational validation would strengthen these results. As backward trajectories primarily indicate the pathways of air masses rather than direct source regions, their interpretation should be made with caution, particularly in cases where air masses may have passed through regions with lower pollutant concentrations that could dilute the observed levels. Therefore, the results of this study are interpreted as supportive evidence, and more precise source apportionment methods, such as the Weighted Potential Source Contribution Function (WPSCF), are suggested for future applications.

## 5. Conclusions

This study analyzed PM_2.5_ concentrations and chemical composition in the Dangjin region from August 2020 to March 2024. The average PM_2.5_ concentration was 26.22 ± 15.29 µg/m^3^, with the lowest seasonal mean in summer (15.68 µg/m^3^) and the highest in winter (30.83 ± 16.31 µg/m^3^).

Among chemical components, NO_3_^−^ was the dominant contributor (5.69 ± 7.02 µg/m^3^, 29.7% of total PM_2.5_), followed by OC (28.3%), SO_4_^2−^ (15.3%), and NH_4_^+^ (15.3%), with secondary inorganic ions together accounting for 60.3% of the mass. The NO_3_^−^/SO_4_^2−^ ratio (1.94) suggested a stronger influence from mobile sources, while the OC/EC ratio (8.72) indicated a substantial contribution from secondary organic carbon.

During high-concentration events (>75 µg/m^3^), levels of NO_3_^−^, NH_4_^+^, SO_4_^2−^, and OC increased significantly. NH_4_^+^ showed the greatest enhancement, up to 6.4-fold in Case 3 (winter), while NO_3_^−^ increased up to 5.3-fold. These events coincided with stagnant meteorological conditions, reduced mixing-layer heights, and enhanced wintertime fuel combustion.

The backward trajectory analysis revealed that high-concentration events were driven by both long-range transported air masses and domestic emissions. In Case 1 (spring), air masses from the northern continental regions were associated with elevated PM_2.5_ levels. Case 2 (winter) reflected the combined effects of domestic heating and industrial activities under stagnant conditions, together with regional-scale inflow from the northeast. In Case 3 (winter), air masses from eastern continental regions and local stagnation contributed to the most severe pollution episode.

Unlike previous studies that focused on short-term observations or limited chemical species, this work provides multi-year data with detailed chemical composition, offering new insights into the combined effects of domestic emissions and transboundary transport.

This study enhances understanding of the seasonal characteristics of PM_2.5_ and the interrelationships among its primary components, elucidating the roles of mobile and stationary sources during high-concentration episodes. In addition, by examining the effect of seasonal meteorological conditions on PM_2.5_ levels, this study underscores the need for airflow analysis and careful consideration of emission sources when formulating air pollution management strategies and policies. As major chemical components of fine particulate matter (ions, organic carbon, and trace elements) can present potential health risks, systematic measures to reduce exposure during high-concentration periods are essential. In particular, the observed concentrations in this study frequently exceeded the World Health Organization (WHO) air quality guidelines, indicating potential risks for adverse health outcomes. A preliminary risk consideration highlights that sustained exposure to elevated levels of nitrates, sulfates, and carbonaceous species may increase respiratory and cardiovascular disease burden in the affected population. Enhancing the management of stationary emission sources (industrial complexes) and implementing complementary policies to reduce emissions from mobile sources (vehicle exhaust) are expected to effectively mitigate high-concentration events. Future research should include more precise source apportionment and long-range transport modeling to better elucidate the contributions of pollutants at the regional level.

## Figures and Tables

**Table 1:** Concentration of substances in the Dangjin area.

Substance (Unit)		N	Mean	S.D.	Max	*p*-Value ^a^
PM_2.5_ (μg/m^3^)		245	26.22	15.29	95.31	0.047
Ion (μg/m^3^)	Cl^−^	245	0.66	0.68	4.91	0.000
	NO_3_^−^	245	5.69	7.02	44.29	0.058
	SO_4_^2−^	245	2.90	2.12	14.18	0.002
	Na^+^	245	0.37	0.36	3.23	0.000
	NH_4_^+^	245	2.82	2.93	23.08	0.023
	K^+^	209	0.27	0.27	3.00	0.000
	Mg^2+^	127	0.11	0.08	1.00	0.078
	Ca^2+^	245	0.13	0.13	0.80	0.000
Carbon (μg/m^3^)	EC	280	0.64	0.29	2.09	0.060
	OC	280	5.58	2.95	16.62	0.448
Elements (ng/m^3^)	Al	51	324.74	627.24	3680	0.027
	Ti	245	8.31	12.23	140.61	0.000
	V	245	1.10	1.22	6.48	0.000
	Mn	245	17.12	15.48	78.68	0.000
	Fe	245	193.80	186.70	1549.08	0.000
	Ni	245	1.54	1.91	20.09	0.000
	Co	245	1.01	1.04	8.22	0.000
	Cu	245	7.27	7.68	42.44	0.000
	Zn	245	62.24	51.21	234.06	0.000
	As	225	3.28	4.95	37.00	0.000
	Sr	245	0.65	1.02	6.33	0.000
	Mo	140	1.35	1.98	12.00	0.000
	Cd	245	1.82	3.34	22.09	0.000
	Ba	245	5.36	7.06	39.82	0.000
	Pb	245	23.31	49.52	621.90	0.003
	P	245	5.69	8.32	54.80	0.000
	S	245	928.19	962.45	4909.62	0.000
	Cr	245	2.43	2.72	22.85	0.000
	Si	245	302.29	563.26	6458.81	0.000

^a^ ANOVA by sampling years.

**Table 2:** Pearson correlation analysis of chemical components in PM_2.5_.

Spring	NO_3_^−^	SO_4_^2−^	NH_4_^+^	OC	Summer	NO_3_^−^	SO_4_^2−^	NH_4_^+^	OC
SO_4_^2−^	0.51				SO_4_^2−^	–0.15			
NH_4_^+^	0.85	0.67			NH_4_^+^	0.15	0.92		
OC	0.55	0.34	0.62		OC	0.79	–0.07	0.19	
EC	0.47	0.26	0.46	0.78	EC	0.52	0.09	0.27	0.50
Fall	NO_3_^−^	SO_4_^2−^	NH_4_^+^	OC	Winter	NO_3_^−^	SO_4_^2−^	NH_4_^+^	OC
SO_4_^2−^	0.64				SO_4_^2−^	0.74			
NH_4_^+^	0.92	0.73			NH_4_^+^	0.94	0.76		
OC	0.62	0.31	0.54		OC	0.69	0.48	0.62	
EC	0.41	0.26	0.41	0.87	EC	0.32	0.12	0.26	0.72

## Data Availability

Most of the data generated or analyzed during this study are included in this published article and Supplementary Materials. Additional datasets are available from the corresponding author upon reasonable request.

## Supplementary Materials

The following supporting information can be downloaded at https://www.mdpi.com/article/10.3390/toxics13100869/s1. Table S1: Specifications of the measurement device used in this study; Table S2: Ion chromatography analysis equipment and conditions; Table S3: Flame ionization detector analysis equipment and conditions; Table S4: Energy dispersive X-ray fluorescence analyzer and conditions.
