# Supplementary material for: Characteristics of the Chemical Components of PM2.5 in the Dangjin Region, South Korea, and Evaluation of Emission Source Contributions During High-Concentration Events
Source: Toxics. 2025 Oct 13;13(10):869. doi: 10.3390/toxics13100869 (PMC12567869; doi:10.3390/toxics13100869)
Supplement: Supplementary file 1 [file toxics-13-00869-s001.zip › toxics-3873009-supplementary.pdf]

Supplementary Information

Characteristics of the Chemical Components of $PM_{2.5}$ in the Dangjin Region, South Korea, and Evaluation of Emission Source Contributions During High-Concentration Events

Young-hyun Kim [1], Shin-Young Park [1], Hyeok Jang [1], Ji-Eun Moon [1] and Cheol-Min Lee [1,2,*]

[1] Department of Chemical and Environmental Engineering, Seokyeong University, Seoul 02713,
Republic of Korea; rladudgus128@skuniv.ac.kr (Y.-h.K.); tlsdud060900@skuniv.ac.kr (S.-Y.P.); amer1can@skuniv.ac.kr (H.J.); mje0313@skuniv.ac.kr (J.-E.M.)

[2] Department of Nano, Chemical and Biological Engineering, Seokyeong University, Seoul 02713,
Republic of Korea

* Correspondence: cheolmin@skuniv.ac.kr; Tel.: +82-2-940-2924

Table S1 Specifications of the measurement device used in this study

PMS-204(APM Co., Ltd, Korea)	
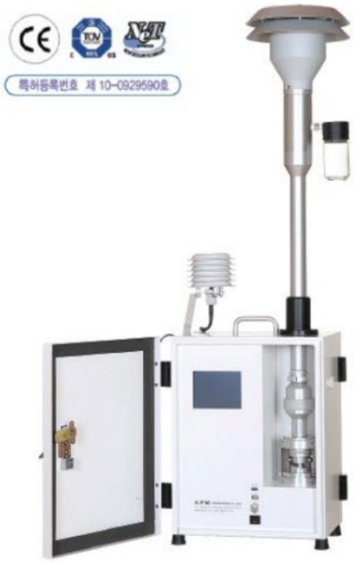	
366(W) × 465(H) × 250(D) MM, 15 kg	
Substance	$PM_{2.5}$

Measurement method	Gravimetrical method
Filter	Teflon (PTFE), Quartz
Operating temperature	–30 ℃–50 ℃
Internal temperature	± 5 ℃
Power	110/220 VAC 50–60 Hz
Flow rate (Flow control accuracy)	0–20 L/min (±1.5% @ F.S)
Memory	USB Memory 4 GB

Table S2 Ion chromatography analysis equipment and conditions

Ion chromatography

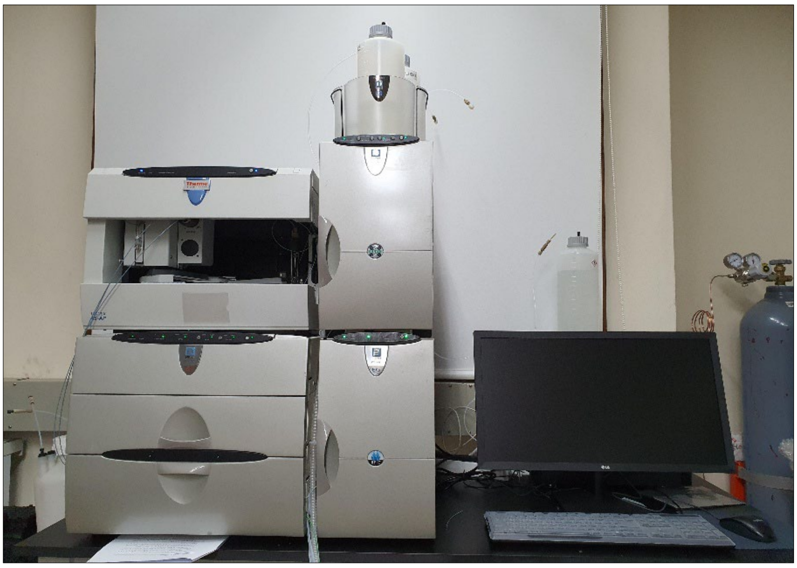

Item	Anion	Cation
Separate column	AS-14	CS-12A
Guard column	AG-14	CG-12A
Suppressor	ADRS 4 mm	CERS 2 mm
Detector	CD 20	CD 20
Flow rate (LPM)	0.5	0.3

Table S3 Flame ionization detector analysis equipment and conditions

Flame ionization detector	
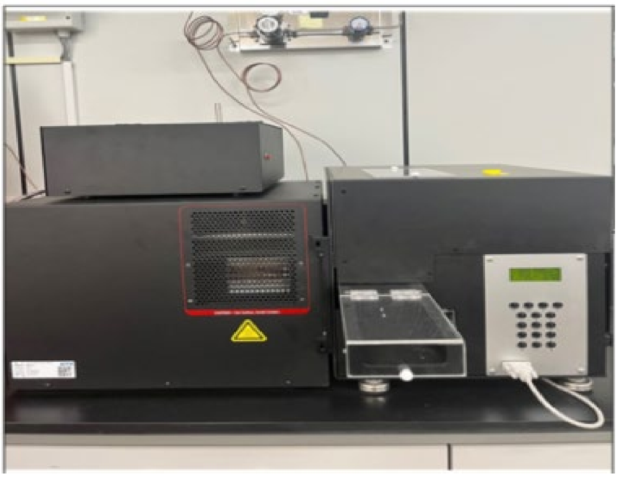	
Detected method	Flame ionization detector
Measuring method	NIOSH 5040, EPA STN Method, User-designed temperature protocols
Analytical techniques	EVOLVED GAS ANALYSIS by thermal-optical analyzer
Analytical components	EC, OC, Carbonate carbon
Measuring range	OC (1–105 µg per filter portion)
Total precision	0.085 at 23 µg/m^3
Precision	0.19 at 1 µg/C, 0.01 at 10 to 72 µg/C
Accuracy	±16.7% at 23 µg/m^3
Expected Load	0.3 µg per filter portion
Operating Characteristics	1) Minimum Quantifiable total OC-0.1 µg/C<br>2) Minimum Quantifiable total EC-0.1 µg/C<br>3) Maximum instrument blank contribution-0.1 µg/C

Table S4 Energy dispersive X-ray fluorescence analyzer and conditions

Energy dispersive X-ray fluorescence analyzer	
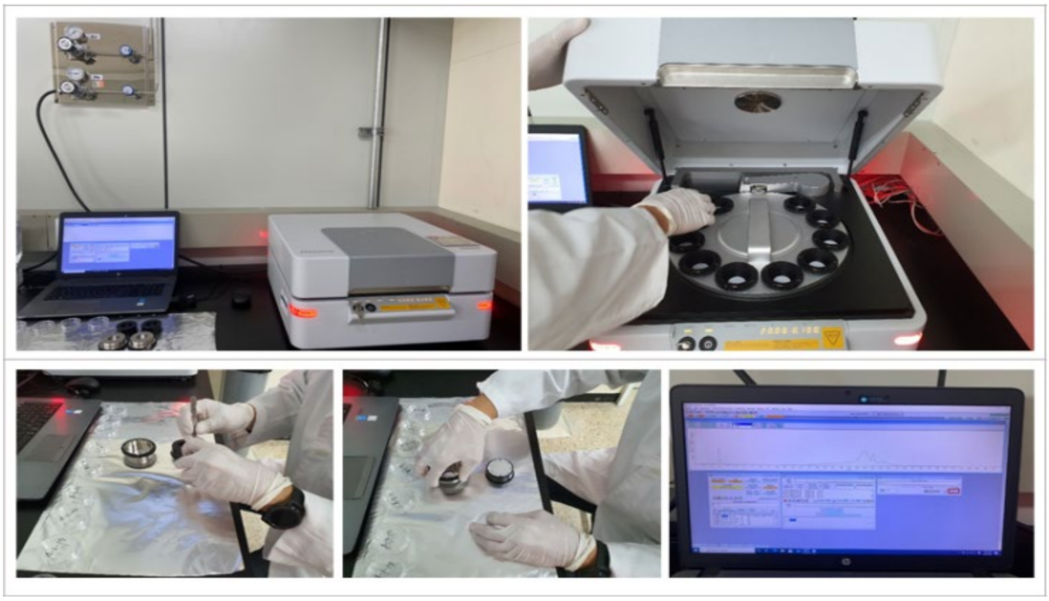	
X-ray optics	-
Composed of 80° of the X-ray tube and detector	Yes
Distance between the SDD detector and sample	<15 mm
Distance between X-ray tube target and sample	<40 mm
X-ray tube	-
End-window type with Rh target	End window Ag target, 15 W
50 µm Be window, air-cooled	Yes
X-ray generator	-
Voltage range	4–50 kV
Current range	0–3.0 mA
Activated area of 30㎟, 20㎟ collimated	Yes

Resolution	140eV@Mn Ka 100 kcps
Maximum count rate	1.5 Mcps
Window	8 µm (0.315 mil)<br>Beryllium
